# Proteomic Analyses of Thioredoxins *f* and *m Arabidopsis thaliana* Mutants Indicate Specific Functions for These Proteins in Plants

**DOI:** 10.3390/antiox8030054

**Published:** 2019-03-02

**Authors:** Juan Fernández-Trijueque, Antonio-Jesús Serrato, Mariam Sahrawy

**Affiliations:** 1 Master Diagnóstica, Avenida del Conocimiento, 100. P.T. Ciencias de la Salud, 18016 Granada, Spain; trijuek@gmail.com; 2 Departamento de Bioquímica, Biología Molecular y Celular de Plantas, Estación Experimental del Zaidín, Consejo Superior de Investigaciones Científicas, C/Profesor Albareda 1, 18008 Granada, Spain; aserrato@eez.csic.es

**Keywords:** thioredoxins, plastidial, specificity, function, proteomic, photosynthesis, Calvin cycle

## Abstract

A large number of plastidial thioredoxins (TRX) are present in chloroplast and the specificity versus the redundancy of their functions is currently under discussion. Several results have highlighted the fact that each TRX has a specific target protein and thus a specific function. In this study we have found that in vitro activation of the fructose-1,6-bisphosphatase (FBPase) enzyme is more efficient when *f1* and *f2* type thioredoxins (TRXs) are used, whilst the *m3* type TRX did not have any effect. In addition, we have carried out a two-dimensional electrophoresis-gel to obtain the protein profiling analyses of the *trxf1*, *f2*, *m1*, *m2*, *m3* and *m4* Arabidopsis mutants. The results revealed quantitative alteration of 86 proteins and demonstrated that the lack of both the *f* and *m* type thioredoxins have diverse effects on the proteome. Interestingly, 68% of the differentially expressed proteins in *trxf1* and *trxf2* mutants were downregulated, whilst 75% were upregulated in *trxm1*, *trxm2*, *trxm3* and *trxm4* lines. The lack of TRX *f1* provoked a higher number of down regulated proteins. The contrary occurred when TRX *m4* was absent. Most of the differentially expressed proteins fell into the categories of metabolic processes, the Calvin–Benson cycle, photosynthesis, response to stress, hormone signalling and protein turnover. Photosynthesis, the Calvin–Benson cycle and carbon metabolism are the most affected processes. Notably, a significant set of proteins related to the answer to stress situations and hormone signalling were affected. Despite some studies being necessary to find specific target proteins, these results show signs that are suggest that the *f* and *m* type plastidial TRXs most likely have some additional specific functions.

## 1. Introduction

Thioredoxins (TRXs) are small proteins (12–14 kDa) present in every organelle with the canonical redox active site WC(G/P)PC and a conserved tertiary structure, which modify their target proteins through the post-translationally reduction of disulphide bonds [1,2]. In the chloroplast, the ferredoxin/thioredoxin system (FTS), composed of ferredoxin (Fdx), ferredoxin thioredoxin reductase (FTR), and TRX, is responsible for the reduction of target proteins involved in a wide range of processes [3]. TRXs have been classified into different groups depending on their primary structures, biochemical properties, and sub-cellular localizations. So far, about 20 TRX types have been identified in plants [4]. This diversity suggests a functional specificity for the different isoforms present in plants, rather than a redundancy. For many years, the best-known plastid TRXs have been of the *f* and *m* type [5,6,7,8]. The *Arabidopsis thaliana* genome contains two TRX *f* (TRX *f1* and *f2*) and four TRX *m* (TRX *m1*, *m2*, *m3* and *m4*). One of the most important biological processes in chloroplasts, the Calvin–Benson cycle, is controlled by TRX *f*, with the reduction mechanism of the fructose-1,6-bisphosphatase (cFBPase) being well known [9]. On the other hand, the *m* type TRXs, originally described as reducers of the malate dehydrogenase (MDH), are more related to photosynthesis [10,11,12]. However, in recent years, many other processes in the chloroplast, such as starch metabolism, photosynthetic electron-transport chain, oxidative-stress response, lipid biosynthesis, nitrogen metabolism, protein folding, and translation [13,14] have been highlighted to be regulated by plastid TRXs. Additionally, other studies have shown evidence of new roles for the plastidial TRXs in heterotrophic tissues, such as roots or flowers [15]. Therefore, it is evident that the importance of the redox regulation through thiol/disulphide interchanges mediated by the thioredoxins happens in almost all the processes of these organelles. For several years, numerous studies have focused on plastidial TRXs, however, from a functional point of view, the information is rather scarce and the debate regarding functional specificity versus redundancy is still open. Technical advances in the coming years will probably allow us to discover many other target proteins.

In order to identify specific functions, the extended use of mutated TRXs for the in vitro target search has proved to be a powerful method that has generated valuable knowledge [16]. However, as we were unable to preserve the in vivo conditions which avoid non-specific TRX-target interactions, the sequence similarity shown among TRX isoforms represents a clear disadvantage for the study of functional specificities. Therefore, to shed more light on possible functional specificities of plastid TRXs we have carried out a novel approach consisting of a wide protein-profiling analysis of Arabidopsis *trxf1, trxf2, trxm1, trxm2, trxm3* and *trxm4* knock-out/down mutants compared with the wild-type plants Columbia 0 (Col0) and Landsberg erecta (Ler). Despite this, more specific studies are necessary, our results suggest that the plastid TRXs we have analyzed are more functionally specialized than expected as we go on to describe in this paper.

## 2. Materials and Methods

### 2.1. Plant Material and Growth Conditions

*Arabidopsis thaliana* wild type plants (ecotype Columbia (Col0) and Landsberg erecta (Ler)), *trxf1* (SALK line SALK_128365), *trxf2* (Gabi-kat line GK_020E05), *trxm1* (SALK line SALK_087118), *trxm2* (SALK line SALK_130686), *trxm3* (ET_3878, background Ler), *trxm4* (SALK line SALK_032538) were grown in soil in a growth chamber at 22 °C under long-day conditions (16 h light/8 h dark) and with photosynthetically active radiation of 120 µmol photons m^−2^ s^−1^. The observed phenotypes, described previously by [17], similar to the wild type lines, were caused by the disruption of the TRX genes. Rosettes from 25 day-old plants were immediately transferred to liquid nitrogen before storage at −80 °C. Expression level was performed by semiquantitative polymerase chain reaction (PCR) analysis using specific oligonucleotides (Table S1) following instructions of Barajas et al. [15].

### 2.2. Protein Extraction, Solubilisation

Protein extraction from a pool of a minimum of 6 rosette plants was performed by using trichloroacetic acid TCA–acetone–phenol protocol [18]. The final pellet was suspended in 600 μL of protein solubilization buffer (9 M urea, 4% 3-[(3-Cholamidopropyl)dimethylammonio]-1-propanesulfonate hydrate (CHAPS), 0.5% TritonX100, and 100 mM dithiothreitol (DTT)). Protein content was quantified by the method of Bradford [19], using bovine serum albumin (BSA) as standard. Three technical replicates of the quantified protein were performed per sample [20].

### 2.3. Isoelectrofocusing, 2-D Electrophoresis, Gel Staining, Image Capture and Analysis

IEF, 2-D electrophoresis, gel staining, image capture, protein spot digestion and MALDI (Matriz-Assisted Laser Desorption/Ionizacion)-TOF (Time of flight) were analyzed in the Universidad of Córdoba UCO-SCAI proteomics facility (Córdoba, Spain), a member of Carlos III Networked Proteomics Platform, ProteoRed-ISCIII. The methodology of Soto et al. was followed [19] as below. Isoelectrofocusing (IEF) was carried out on Precast 17 cm IPG pH 5–8 linear gradient (Bio-Rad, Hercules, CA, USA) strips. The strips were allowed to rehydrate in a PROTEAN IEF Cell (Bio-Rad) for 14–16 h at 50 V and 20 °C with 315 μL of protein solubilization buffer containing 400 μg of proteins. The proteins were separated in the pH range 4–7 by using IEF in three-step procedure as follow, 15 min at 500 V, followed by 2 h at 10,000 V and a final step of 10,000 Vh to complete 60,000 Vh. The strips were immediately run after focusing. The strips were equilibrated after immersion for 20 min first in 375 mM Tris–HCl, pH 8.8, with 6 M urea, 2% sodium dodecyl sulfate (SDS), 20% glycerol, and 2% DTT, and then in the same solution containing 2.5% iodoacetamide as a substitute of DTT. After transferring the strips onto vertical slab 12% SDS-polyacrylamide gels (Bio-Rad PROTEAN Plus Dodeca Cell), the electrophoresis was run at 55 mA/gel until the dye front reached the bottom of the gel [20]. The gels were silver-stained as described by Yan et al. [21] or with coomassie brilliant blue G-250 (Sigma, (Sigma-Aldrich Chemical Co, St Louis, MO, USA). Gel images were captured, digitalized (Molecular Imager Pharos FX^TM^ Plus multi Imager System, Bio-Rad), and analyzed with PDQuest^TM^ 2-D analysis software (Bio-Rad laboratories, Hercules, CA, USA), and as a minimum criterion for presence/absence, ten-fold over background was used. Significant spots were excised automatically using *ProPic*) (Genomics Solutions Inc., Ann Arbor, USA), stored in milli-Q water until Matrix-Assited Laser Desorption/Ionization—Time-Of-Flight (MALDI-TOF/TOF) analysis. The protocols for digestion were performed as described previously [22]. For the MAILDI-TOF analysis, a combined Peptide Mass Fingerprinting (PMF) search Mass Spectrometry (MS plus MS/MS) was performed using GPS Explorer^TM^ software v 3.5 (Applied Biosystems, Waltham, Massachusetts, MA USA) over non-reductant NCBInr database using the MASCOT search engine (Matrix Science, London; http://www.matrixscience.com) following parameters reported previously [22].

## 3. Results

### Differentially Regulated Proteins from Rosettes of trxf1, trxf2, trxm1, trxm2, trxm3 and trxm4 Mutants

**Figure 1:**
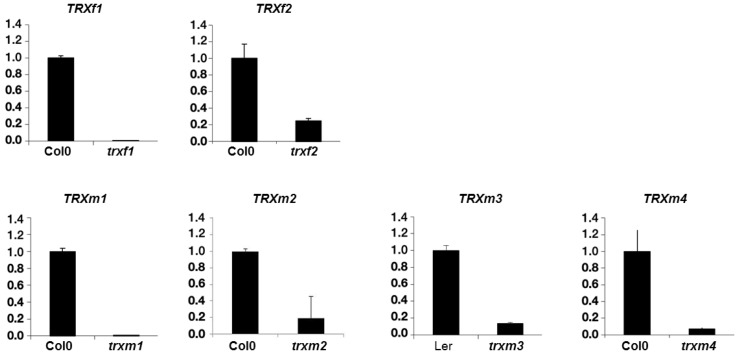
Thioredoxins (TRXs) *f* and *m* transcript levels in *trxf* and *trxm* mutants.

**Figure 2:**
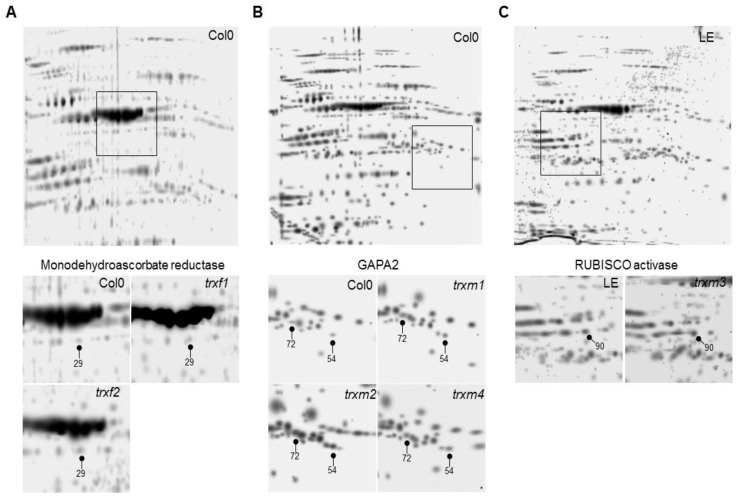
DE images from rosette of *trxf1, f2, m1, m2, m3* and *m4* mutants. 2-D images of total proteins from rosettes of *trx f1* and *f2* (**A**)*, m1, m2* and *m4* (**B**), and *m3* (**C**) mutants in comparison with Col0 or Ler (LE). Numbers correspond to the protein spots identified by MALDI-TOF/TOF analysis (Table S2). The figure shows the representative experiments carried out with some examples of proteins identified in each gel.

**Figure 3:**
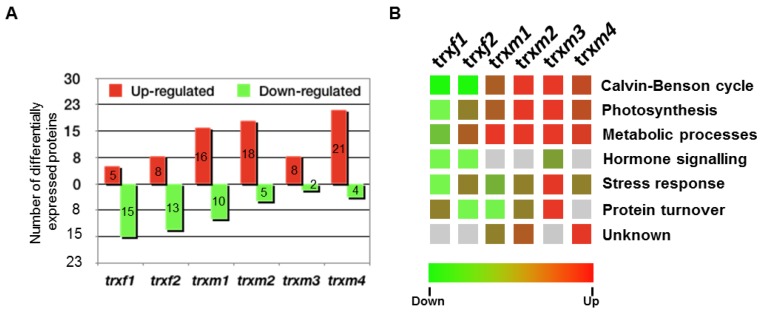
Processes down- or up-regulated in the *trx* mutants, number of coincident spots identified by proteomic analyses. (**A**) number of proteins down- or up-regulated in *trxf* or *trxm* mutants; (**B**) biological processes affected in each mutant line.

The *trxm1* and *trxm4* mutants were described previously [23,24] as well as *trxm3* [25], *trxf1* [26,27] and *trxf2* [26]. Figure 1 shows that there is no detected TRX expression in *trxf1*, *trxm1*, *trxm3* and *trxm4*, with these lines being considered as knockout ones, whilst a slight level of transcripts can be observed for *trxf2* and *trxm2* as it was described. The level of expression of all the mutants was clearly sufficiently low to validate the results obtained. The proteomic approach was performed to study protein profiles in these plastidial TRX *f* and *m* mutants. For this, the total protein extracts from rosettes of 25 dpg plants of the mutant lines *trxf1*, *trxf2*, *trxm1*, *trxm2*, *trxm3* and *trxm4* and of the wild type lines Col0 and Ler were analyzed in the “Unidad de Proteómica of the Universidad of Córdoba” (Córdoba, Spain). An optimal concentration and purity degree of the protein extracts were reached for a high level of separation of the peptides using two dimensional (2-DE) electrophoresis. Figure 2 shows the spots corresponding to the peptides of up- and down-regulated proteins in rosettes from *trxf1* and *trxf2* (Figure 2A), *trxm1*, *trxm2* and *trxm4* (Figure 2B), and *trxm3* (Figure 2C), mutants in comparison with Columbia (Col0) and Landsberg erecta LE (Table 1, Table 2 and Table 3. The master gels from each three replicates gel mutant lines were obtained and normalized by using the software PDQuest^®^ (Bio-rad). Three replicates gel of Col0 control was developed for each TRX type (*f* and *m*), as Ler for *trxm3* (Figure 2). Also, PDQuest^®^ (Bio-rad) was used to select, in each gel of the mutant lines, those spots where the expression was down regulated or up regulated when compared to the same spot located in the gels of the control plants. The proteins contained in the spots were picked, digested and the peptides identified by MS (Table S2). The comparison with databases allowed us to identify the proteins that could contain the different peptides. Out of the 200 analyzed and resolved spots, a total of 86 differentially expressed proteins were identified (Table S2). The *trxf1* and *trxf2* mutants had a proportionally larger number of down regulated proteins (15 out of 20 and 13 out of 21, respectively), whilst the *trxm* mutants had a larger number of up regulated proteins (16 out of 26 in *trxm1*, 18 out of 23 in *trxm2*, eight out of 10 in *trxm3*, and 21 out of 25 in *trxm4*) (Figure 3A). Despite the sequence similarities between the TRX *f1* and *f2*, in the *trxf1* and *trxf2* lines nine proteins showed a different regulation (Table 1): glutamate-glyoxylate aminotransferase 1 (GGAT1), aminomethyltransferase, 5-methyl tetrahydropteroyl triglutamate-homocysteine methyltransferase 1, ribulose-bisphosphate carboxylase oxygenase (RUBISCO) activase, β-D-glucopyranosyl abscisate β-glucosidase, glyceraldehyde-3-phosphate dehydrogenase (GAPC2), monodehydroascorbate reductase, V-type ATP synthase, and Chaperonin 60 subunit β1. Nevertheless, apart from these differences, 10 out of 24 (41.7%) of the analyzed proteins were down regulated in both mutant lines while four out of 24 (16.7%) were up regulated. Data analysis revealed that the largest number of down regulated biological processes corresponded to the *trxf* mutants (Figure 3B). The other *m* type TRXs mutants mostly showed up regulated processes, especially *trxm2, trxm3* and *trxm4* (Figure 3B).

The four *trxm* mutant lines only shared one differentially expressed protein (β carbonic anhydrase 2), while the *trxm1*, *trxm2*, and *trxm4* lines shared up to 13 differentially expressed proteins (39.4%, Table 2 and Table 3). However, some of the differentially expressed proteins identified were up regulated in one mutant line but down regulated in another one, as in the case of the ferredoxin-NADP reductase 1 or the chlorophyll a–b binding protein 2. Interestingly, nine proteins underwent similar changes in these three *trxm* lines, eight up-regulated and only one down-regulated (ferredoxin-NADP reductase 2). Some interesting proteins up-regulated, affecting key pathways, were the two chloroplast isoforms of glyceraldehyde-3-phosphate dehydrogenase GAPB and GAPA2 and the mitochondrial enzymes serine hydroxymethyltransferase 1 and glycine dehydrogenase 1 (amino acid metabolism). Regarding carbon fixation, the RUBISCO large subunit was down regulated in *trxm1* and up regulated in *trxm4*, whereas its regulator RUBISCO activase was up-regulated in *trxm2*, *trxm3,* and *trxm4.* Interestingly, no photosynthetic genes were differentially expressed in the *trxm3* mutant; nevertheless, the up-regulation of photosystem II stability/assembly factor HCF136 in the *trxm1*, *trxm2* and *trxm4* mutant lines is significant.

No coincident protein was observed in the *trxf* and *trxm* mutants. Nevertheless, the *trxf1* and *trxf2* lines shared one differentially expressed protein with the *trxm1*, *trxm2* and *trxm4* mutants: the ferredoxin-NADP reductase 1, though this protein was up-regulated in *trxf1*, *trxf2*, and *trxm4* and down-regulated in *trxm1* and *trxm2*. In addition, in the *trxf* and *trxm3* mutants only myrosinase 2, a ABA signalling protein, was differentially expressed (down-regulated).

Regarding hormonal processes, in the *trxm* mutants only *trxm3* displayed changes in ABA and JA signalling. Adjustments in the *trxf* mutants were limited to ABA signalling with a down-regulation of myrosinase 2 in both mutant lines and of β-D-glucopyranosyl abscisate β-glucosidase (activating the glucose-conjugated inactive ABA), but only in the *trxf1* mutant. In Table 1, Table 2 and Table 3, we can observe the metabolic alterations mostly corresponded to changes in the level of glycine, alanine, glutamine, and methionine synthesizing/hydrolyzing enzymes in chloroplasts, mitochondria, peroxisomes, and cytosol. However, we could not observe any change in the amino acid metabolism in the *trxm3* mutant.

**Figure 4:**
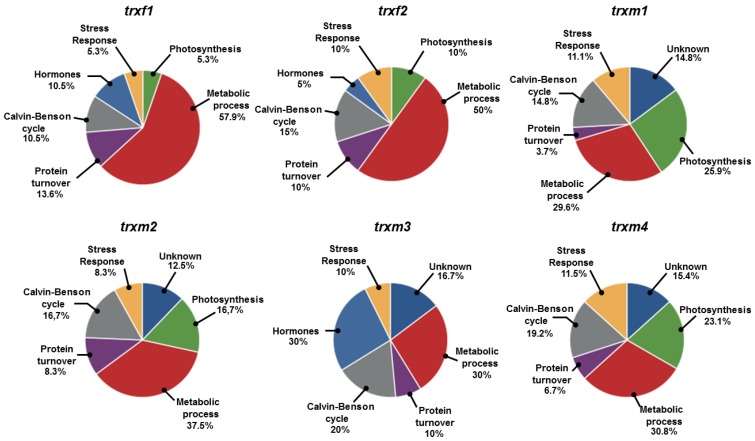
Degree of significance of the biological processes affected in the *trx* mutants in relation to the number peptides predicted to be involved in a process.

All the identified spots corresponded to proteins with putative general functions related to the Calvin–Benson cycle, photosynthesis, stress response, hormone signalling, protein turnover, and to unknown processes (Figure 4, Table 1, Table 2, Table 3 and Table S2). The most represented functions fell into the metabolism category, containing 30–58% of the spots analysed, especially in the *trxf* mutants. The Calvin–Benson cycle was also well represented in all the mutants, ranging from 10.5% to 20%. Surprisingly, photosynthesis was affected in all the mutants except for *trxm3*, especially in the *trxm1*, *trxm2*, and *trxm4* lines (Figure 4).

**Figure 5:**
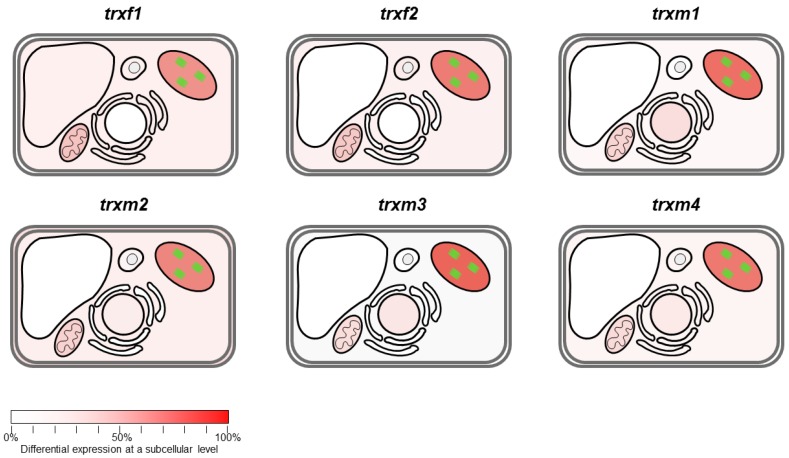
Differential expression shown at a sub-cellular level. The red color intensity is indicating if differential expressions are significant in a given organelle.

**Figure 6:**
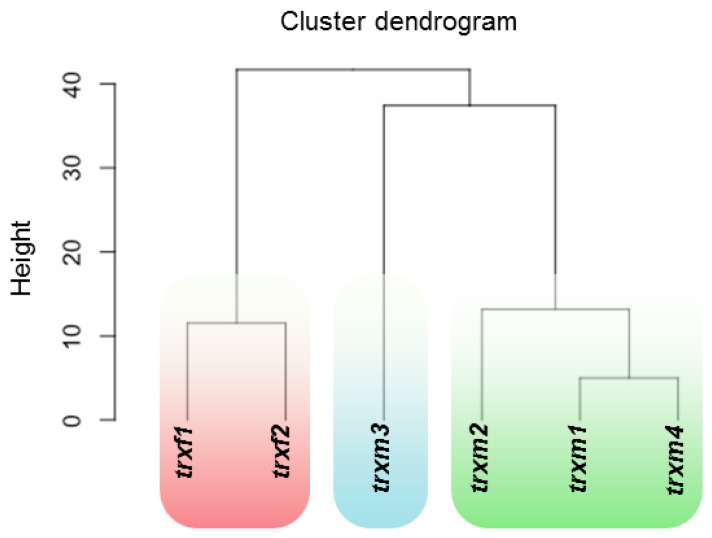
Hierarchical clustering segregates *trx* mutants according to the biological processes affected. For calculations, the R statistical environment (The R Foundation, Vienna, Austria) available at http://www.R-project.org was used.

At a sub-cellular level, most of the differential expressed proteins were predicted to be chloroplastic, ranging from 47% to 59% of the total spots identified in each mutant (Figure 5). Differential expression in TRX mutants also affected non-chloroplast proteins as they were also predicted to be located in other sub-cellular compartments such as mitochondria, peroxisomes, cytosol, nucleus, Golgi/endoplasmic reticulum, and vacuole (enumerated according to the frequency of appearance in the proteomic analyses). Taking into account the affected processes, we applied a hierarchical clustering to our proteomic data (Figure 6). Two major clusters contained the *trxf* and the *trxm* mutants. Within the *trxm* cluster, two sub-clusters separated the *trxm3* line from the *trxm1*, *trxm2*, and *trxm4* mutants. Interestingly, according to our clustering analysis, *trxm1* might be functionally closer to *trxm4* than to *trxm2*. These data are according to recent results obtained in our laboratory (unpublished data).

## 4. Discussion

The existence of an elevated number of plastidial thioredoxins, amongst which are two *f* type TRXs and four *m* type TRXs in the chloroplast makes it difficult to identify and separate their different functions. A great deal of effort has been made to reach important conclusions and different authors have reported previously protein targets and suggested specific roles for several of the plastidial TRXs. Due to similarities with the *f* and *m* type TRXs, these isoforms might both carry out overlapping as specific functions in the redox regulation of the plant physiological processes. However, it has been difficult to find a clear border to delimit the role of each plastidial TRX described up to now. Beyond the study of the different reduction degree of the target proteins, our aim has been to know whether the pattern response of the Arabidopsis proteome is specific for each plastidial TRX mutant characterized. In this study we have carried out a proteomic profile of Arabidopsis mutant lines lacking *f1*, *f2*, *m1*, *m2*, *m3* and *m4* type TRXs.

The analysis of the 2-DE pattern revealed a total of 86 different protein-spot intensities out of the 200 that were resolved in all images (Figure 2 and Table S2). The differentially expressed proteins in the mutants showed particular patterns, supporting the hypothesis of a specific functionality for each TRX *f* or *m* isoform. A higher number of down regulated proteins were observed for *trxf* mutants, mainly *trxf1*; whilst more up regulated proteins occurred in the *trxm* lines, principally the *trxm4* line (Figure 3A), suggesting that their roles are separate and affected.

Almost 59% of the differentially expressed proteins are plastidial, but other affected proteins are localized in mitochondria, cytosol, peroxisomes and nucleus (Figure 5), indicating the connection between the processes occurring either in the chloroplast or in other localizations when one plastidial TRX is missing. Likely, redox imbalance provoked by the lack of TRXs *f*/*m* isoforms in chloroplasts is triggering retrograde signalling to readjust the biological functions affected as we noticed differentially expressed proteins in other organelles. Moreover, we cannot discard other factors regulating the protein levels as protein degradation or stabilization by proteases and chaperonins, respectively, as discussed later.

As expected for the chloroplast redox enzymes involved in photosynthesis-related processes, among the proteins found that predominate are those which are functionally related with electronic transport. The presence of several subunits of the chloroplastidic and mithocondrial ATP synthase complexes is noteworthy. The subunit α of the chloroplastidic ATP synthase was down regulated in the *trxf1* and *trxf2,* whilst the subunit β was up regulated in these lines. Interestingly, the subunits β and γ (the latter being up-regulated in *trxm3*) have been reported to be targets of plastidial TRXs [1,28]. However, it has been described that under low irradiance, NADPH thioredoxin reductase C (NTRC) is required for the redox modulation of the subunit γ of ATP synthase [29]. As the subunit 1 of the mitochondrial ATP synthase is also affected in *trxf1*, *trxf2* and *trxm3* mutants, these patterns of up/down regulation would be an indication of the importance and complexity of the regulation of ATP synthesis in plants suggesting the significance of balancing chloroplastic and mitochondrial ATP levels in response to redox changes. Remarkably, and similarly to the above-mentioned chloroplast subunits, the mitochondrial subunit 1 also seems to be redox regulated [1,30].

The link between chloroplast and mitochondrion is also evidenced with proteins responding to redox changes in the chloroplast involved in amino acid metabolism, such as serine hydroxyl methyl transferase or glycine dehydrogenase in *trxm1*, *trxm2* and *trxm4* and amino methyl transferase in *trxf1*; and even proteins with unknown functions such as the protein with identity number B3H6G1 in the *trxm1*, *trxm2* and *trxm4* mutants (Table S2).

These results do not necessarily imply dual localization chloroplast/mitochondria of the referred isoforms (though this cannot be ruled out), but rather they demonstrate the relationship between the metabolism of the chloroplast and the mitochondria. The observed differences at a proteomic level reveal, once again, a certain degree of functional specificity of each isoform of analyzed plastidial TRX.

Metabolism, the Calvin–Benson cycle, photosynthesis, response to stress, and hormone signaling are the functional categories most affected when one of the *f* or *m* type TRXs is absent. However, the lack of TRX *f1* or *f2* provokes a down-regulation in cell general processes, whilst the influence of the absence of TRXs *m* is the opposite (Figure 3). Most of the proteins differentially expressed in the *trxf* mutants relate to its function in metabolism in general, and to the synthesis of ATP specifically. In the same way, it seems that TRXs *m1, m2* and *m4* are involved in the redox regulation of the photosynthesis. TRXs *f* and *m3* are likely to be involved in the redox regulation during stress situations as monodehydroascorbate reductase (in the stress response category) and myrosinase 2 (in the ABA signaling category) have been reported as TRXs targets (Table 1 and Table 3).

Taking into consideration the data obtained, it seems that the protein differentially expressed in the mutants fell into different functional categories which can be organized in function of the processes affected or their sub-cellular localization. Thus, as expected in a photosynthetic organ like the leaf, among the predominate proteins found are those which are functionally related with electronic transport with the only exception of *trxm3*.

Likewise, photosynthesis appears highly unstable in these mutants, amongst which a number of the electron transport or Calvin-Benson cycle proteins are up regulated or down regulated, as is described in the Results section. Interestingly, factor HCF136 was up regulated in the *trxm1*, *trxm2* and *trxm4* mutant lines, but down regulated in the *trxf1* and *trxf2* lines. It has been reported that factor HCF136 is involved in the assembly of an intermediary complex of PSII [31], suggesting that it is possible to detect putative alterations or increased instability of PSII in these mutants.

Previous studies have reported that TRXs are able to transfer reducing equivalents to the redox protein HCF164 in the thylakoid lumen [32], and that these proteins are necessary for the biogenesis of PSI. Additionally, evidence showed that HCF164 serves as transducer of reducing equivalents to proteins in the thylakoid lumen. Consequently, decreased levels of *m* type TRXs might lead to a redox imbalance in the thylakoid lumen and the instability of redox regulated proteins components of the PSII as PsbO subunits [14]. Interestingly, PSI subunit levels do not seem to be influenced in the TRX mutants analyzed, ruling out the possibility of different spot intensities (detection threshold) as both photosystems have a similar stoichiometry in the thylakoid membranes. Curiously, apart from proteins participating in primary metabolism or photosynthesis, other less represented proteins did not appear in our analyses, suggesting that these spots were not abundant enough to be detected with our experimental conditions. A clear example is the absence among the identified spots of the plastid TRXs that we were analyzing. However, this fact does not invalidate our results.

Some authors have attributed the *f* and *m* type TRXs to being part of the mechanisms involved in the control of the binding of the light harvesting chlorophyl (LHC) antenna complexes to the PSI and PSII through the activation of a serine/threonine kinase, SNT7 [33]. This is relevant because two components of binding factors to the chlorophyll *a/b* of the PSII are differentially expressed in *trxm1* (Lhcb1B2 is up regulated and Lhcb2C1 is down regulated), *trxm2* (Lhcb1B2 is up regulated) and *trxm4* (Lhcb2C1 is up regulated). Additionally, *trxm1* was the only one to have differentially expressed two subunits of the photolysis water complex (PsbO2 is up regulated and PsbP1 is down regulated), essential for generating the reducing power during the electron transport chain. TRX *m1* is probably closely related to the redox regulation of the photosynthesis process.

In relation to the carbon fixation, it was not surprising to detect the large subunit of the RUBISCO protein among the differentially expressed proteins, due to its relevant position in carbon fixation. The RUBISCO large subunit was found to be down regulated in all the *trxf* and *trxm1* lines. However, we cannot rule out the possibility that these changes might correspond to post-translationally modifications of the RUBISCO large subunit as we did not observe differences in the phenotypes with respect to wild-type plants due to a putative defect in carbon fixation. RUBISCO activase, involved in the regulation of the small subunit of RUBISCO appeared up regulated in the *trxm2*, *trxm3* and *trxm4* lines and down regulated in the *trxf2* mutant, indicating a specific action when a change occurs in CO_2_ fixation during the loss of the redox homeostasis in plants. It is well known that several Calvin-Benson cycle enzymes are redox regulated, so it is not surprising to find them in the proteome of the mutants. However, the list is certainly lower due to the high plasticity of the plant to adapt under different adverse environments.

Due to their importance in key positions in the Calvin-Benson cycle the following proteins are particularly relevant: fructose-bisphosphate aldolase (up regulated in *trxm1*, *trxm2* and *trxm3*) and transketolase 1 (down regulated in *trxf1*, *trxf2* and *trxm1*); and the plastidial isoforms of GADPH, found to be up regulated in *trxm1, trxm2* and *trxm4* mutants; which were confirmed in the gene expression analysis (data not shown). It seems clear that redox activation/regulation by TRXs has a direct effect on the balance of carbohydrate synthesis and distribution as an optimal cell redox homeostasis environment is mandatory for the proper functioning of several processes, essentially carbon metabolism and the photosynthesis.

An interesting group was that of the proteins involved in redox homeostasis, with the most relevant being the subunits 1 and 2 of ferredoxin NADP^+^ reductase (FNR) (not described so far as a target of plastidial TRXs) and chloroplastidic and peroxisomal monodehydroascorbate reductase (MDAR), the relationship between these proteins and the redox regulation through TRXs is well known [34]. One of the possible functions of plastidial TRXs could be to maintain homeostasis against abiotic stress conditions, such as salinity during germination [35]. It seems that *f* type TRXs possibly play a more relevant role in controlling the homeostasis conditions inside the cells than previously thought.

Enzymes were also found to be involved in the response to biotic stress, such as the myrosinase (down regulated in *trxf1*, *trxf2* and *trxm3*). In addition, the epithiospecifier protein, up regulated in *trxm3*, is also functionally related to myrosinase [36]. Similarly, GDSL esterase/lipase endothelial cell specific molecule1 (ESM1) was found to be up-regulated in *trxm1*, *trxm2* and *trxm4* mutants and it has been related to the response to insects [37].

Moreover, several interesting proteins related with the metabolism of amino acids, proteins and lipids have also been identified. Differentially expressed enzymes, mainly related with the synthesis and transfer of amino and methyl groups were found to be up regulated. Concerning the metabolism of proteins, proteases appeared, as well as factors involved in the translation process and proteins with chaperone activity. Particularly Relevant are the chaperonin 60 subunit β1 (up regulated in *trxf1* and down regulated in *trxf2*) and chaperonin 60 subunit β2 (up-regulated in *trxm2* and *trxm3*), suggesting that the protein folding and assembly are likely to be related to redox regulation. Previous studies have shown that the chaperonin 60 subunit β may be protecting the photosynthtetic components during stress situations [38].

Although the number of proteins with unknown functions was very low, a few examples without characterization were found (four), such as the protein with identity number B3H6G1 up regulated in *trxm1*, *trxm2* and *trxm4* (mentioned above). It is reasonable to think that a deep characterization would be necessary to identify new and specific functions of these plastidial thioredoxins.

## 5. Conclusions

In this study we have attempted to shed some light and to get closer to functionally defining the different isoforms of *f* and *m* type TRXs in Arabidopsis and to clarify the blurred frontier that exists, in most cases, between specificity and functional redundancy in multi-gene families in plants. Even though there are numerous studies on plastidial TRXs, the existing information is still scarce, from a functional point of view. Despite this, more studies are necessary, this is the first time a broad view is showing what processes are affected when one of the *f* or *m* type TRXs is lacking. We were aware of the complexity of the task. The results we have achieved from the approach followed in this study, which consisted of a comparative analysis of knockout/down mutants for the six isoforms of *f* and *m* type TRXs, should help to better understand the functional role of *f* and *m* type TRXs in Arabidopsis and to open up new research paths in the study of processes regulated by these enzymes.

## Figures and Tables

**Table 1:** Differentially expressed proteins identified by MS in the *trxf1* and *trxf2* mutants, organized inthe functional category, the gene code and the subcellular localization and whether the protein has been reported as a Thioredoxins (TRX) target. The color code indicates fold change in protein abundance.

Functional Category	Protein	Gene ID	Location	TRX Target	Spot	*trxf1*	*trxf2*
Amino acids metabolism	Glutamate-glyoxylate aminotransferase 1	At1g23310	Per.	-	16		
	Aminomethyltransferase	At1g11860	Mit.	-	8		
	Glutamate-glyoxylate aminotransferase 2	At1g70580	Per.	-	21		
	5-methyltetrahydropteroyltrigluta-mate-homocysteine methyltransferase 1	At5g17920	Per.	-	33		
	5-methyltetrahydropteroyltrigluta-mate-homocysteine methyltransferase 2	At3g03780	Cy.	-	12		
Calvin-Benson cycle	RUBISCO large subunit	AtCg00490	Ch.	Yes	5		
	Transketolase1	At3g60750	Ch.	Yes	20		
	RUBISCO activase	At2g39730	Ch.	Yes	27		
ATP synthesis	ATP synthase subunit ß	AtCg00480	Ch.	Yes	15		
	ATP synthase subunit α	AtCg00120	Ch.	Yes	4, 24		
	ATP synthase subunit 1	AtMg01190	Mit.	Yes	25		
Photosynthesis	PSII stability/assembly factor HCF136	At5g23120	Ch.	-	9		
	Ferredoxin-NADP reductase 1	At5g66190	Ch.	-	17		
ABA signalling	β-D-glucopyranosyl abscisate β-glucosidase	At1g52400	ER	-	3		
	Myrosinase 2	At5g25980	n.d.	Yes	22		
Glycolisis	Triosephosphate isomerase	At3g55440	Mit.	-	31		
	Glyceraldehyde-3-phosphate dehydrogenase C2 (GAPC2)	At1g13440	Cy.	-	26		
Stress response	Jacalin-Related lectin	At3g16470	n.d.	-	7		
	Monodehydroascorbate reductase	At1g63940	Ch., Mit.	Yes	29		
Protein biosynthesis	Elongation factor Tu	At4g20360	Ch.	Yes	6		
ATP hydrolysis	V-type ATP synthase	At1g78900	V.	-	2		
PSII biogenesis	PSII stability/assembly factor HCF136	At5g23120	Ch.	-	30		
Refolding activity	Chaperonin 60 subunit ß1	At1g55490	Ch.	-	13		
Tricarboxylic acid cycle	Malate dehydrogenase 1	At1g53240	Mit.	Yes	35		
Protein abundance change relative to the control (Col0).		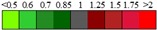					

Ch., chloroplast; Mit., mitochondria; Per., peroxisome; Cy., cytosol; V., vacuole; ER, endoplasmic reticulum; n.d., not determined. Proteins with a Confidence Interval C.I.% ≥ 95% are shown. According to Montrichard et al. (2009) [1], reported thioredoxin targets are shown. NADP: nicotinamide-adenine-dinucleotide phosphate; HCF: high chlorophyll fluorescence; ABA: absicic acid; PSII: photosystem II; RUBISCO: ribulose bisphosphate carboxylase/oxygenase.

**Table 2:** Differentially expressed proteins identified by MS in the *trxm1*, *trxm2*, and *trxm4* mutants, organized in the functional category, the gene code and the subcellular localization and whether the protein has been reported as a TRX target. The color code indicates fold change in protein abundance.

Functional Category	Protein	Gene ID	Location	TRX Target	Spot	*trxm1*	*trxm2*	*trxm4*
Calvin-Benson cycle	Transketolase	At3g60750	Ch.	Yes	38			
	RUBISCO large subunit	AtCg00490	Ch.	Yes	28			
	Fructose-bisphosphate aldolase 2	At4g38970	Ch.	Yes	59, 103			
	RUBISCO activase	At2g39730	Ch.	Yes	65			
	Glyceraldehyde-3-phosphate dehydrogenase B GAPB)	At1g42970	Ch.	Yes	68			
	Glyceraldehyde-3-phosphate dehydrogenase A2 (GAPA2)	At1g12900	Ch.	Yes	54, 72			
Amino acids metabolism	Serine hydroxymethyltransferase 1	At4g37930	Mit.	Yes	101			
	Glycine dehydrogenase (decarboxylating) 1	At4g33010	Mit.	-	100			
	Probable phosphoglycerate mutase 2	At3g08590	Mit.	-	47			
	Glutamate-glyoxylate aminotransferase 1	At1g23310	Per.	Yes	97			
	5-methyltetrahydropteroyltrigluta-mate-homocysteine methyltransferase 1	At5g17920	Cy.	Yes	45, 98			
Photosynthesis	Chlorophyll a-b binding protein 2	At1g29920	Ch.	Yes	42			
	Ferredoxin-NADP reductase 1	At5g66190	Ch.	-	43			
	Chlorophyll a-b binding protein	At2g34420	Ch.	-	56			
	Oxygen-evolving enhancer protein 2-1	At1g06680	Ch.	Yes	57			
	Oxygen-evolving enhancer protein 1-2	At3g50820	Ch.	Yes	41			
	Ferredoxin-NADP reductase 2	At1g20020	Ch.	-	104			
Stress response	Uncharacterized protein	At2g37660	Ch.	-	40			
	Heat shock 70 kDa protein 3	At3g09440	N.	-	46			
	Monodehydroascorbate reductase 1	At3g52880	Per.	Yes	70			
PSII stabilization/repair	Photosystem II stability/assembly factor HCF136	At5g23120	Ch.	-	67			
	Protease Do-like 1	At3g27925	Ch.	-	39			
Protein transport	Chaperone protein ClpC1	At5g50920	Ch.	-	37			
ATP synthesis	ATP synthase subunit beta	AtCg00480	Ch.	Yes	93			
Glycolysis	Glyceraldehyde-3-phosphate dehydrogenase GAPC2	At1g13440	Cy.	Yes	53			
Carbohydrate metabolism	Chloroplast stem-loop binding protein of 41 kDa b	At1g09340	Ch.	-	64			
Carbon utilization	β carbonic anhydrase 2	At5g14740	Ch.	-	55			
Lipid degradation	GDSL esterase/lipase ESM1	At3g14210	N.	-	105			
Protein refolding	Chaperonin 60 subunit beta 2	At3g13470	Ch.	Yes	96			
Unkown	Polyketide cyclase/dehydrase and lipid transport superfamily protein	At4g14500	Mit.	-	61			
	Uncharacterized protein	At2g37660	Ch.	-	40			
	Uncharacterized protein	At5g05113	Mit.	-	74			
	Disease resistance protein (NBS-LRR class) family	At5g40060	n.d.	-	75			
Protein abundance change relative to the control (Col0).		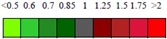						

Ch., chloroplast; Mit., mitochondria; Per., peroxisome; Cy., cytosol; V., vacuole; N., nucleus; n.d., not determined. Proteins with Protein Scores C.I.% ≥ 95% are shown. According to Montrichard et al. (2009) [1], reported thioredoxin targets are shown. ESM1: epithiospecifier modifier 1.

**Table 3:** Differentially expressed proteins identified by MS in the *trxm3* mutant, organized in the functional category, the gene code and the subcellular localization and whether the protein has been reported as a TRX target. The color code indicates fold change in protein abundance.

Functional Category	Protein	Gene ID	Location	TRX Target	Spot	*trxm3*
Calvin-Benson cycle	RUBISCO activase	At2g39730	Ch.	Yes	90	
	Fructose-bisphosphate aldolase 2	At4g38970	Ch.	Yes	91	
ATP synthesis	ATP synthase subunit 1	AtMG01190	Mit.	Yes	82	
	ATP synthase γ chain 1	At4g04640	Ch.	Yes	85	
JA signalling /response	Epithiospecifier protein	At1g54040	N.	-	84	
	Lipoxygenase 2	At3g45140	Ch.	Yes	76	
ABA signalling	Myrosinase 2	At5g25980	n.d.	Yes	77	
Carbon utilization	β carbonic anhydrase 2	At5g14740	Ch.	Yes	92	
Refolding activity	Chaperonin 60 subunit β 2	At3g13470	Ch.	-	87	
Stress response	Monodehydroascorbate reductase	At1g63940	Ch., Mit.	Yes	83	
Protein abundance change relative to the control (Ler).		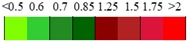				

Ch., chloroplast; Mit., mitochondria; N., nucleus; n.d., not determined. Proteins with Protein Scores C.I.% ≥ 95% are shown. According to Montrichard et al. (2009) [1], reported thioredoxin targets are shown. JA: jamonic acid.

## Supplementary Materials

The following are available online at https://www.mdpi.com/2076-3921/8/3/54/s1, Table S1: gene-specific oligonucleotides used for quantitative PCR, Table S2: list of identified proteins in *trxf1*, *trxf2 trxm1*, *trxm2*, *txm3*, and *trxm4* mutants.
