# Supplementary material for: Proteomic Analyses of Thioredoxins f and m Arabidopsis thaliana Mutants Indicate Specific Functions for These Proteins in Plants
Source: Antioxidants (Basel). 2019 Mar 2;8(3):54. doi: 10.3390/antiox8030054 (PMC6466581; doi:10.3390/antiox8030054)
Supplement: Supplementary file 1 [file antioxidants-08-00054-s001.pdf]

Table S1. Gene-specific oligonucleotides used for semi-quantitative PCR

Oligos	Gene Analyzed	Primer sequence
Atf1a	At3g02730	AAACAGCGAGGTCTGCTGCT
Atf1b		TAACACCAGATTTCACATTACATACAAACA
Atf2a	At5g16400	TCCGTTATTCTCCGATTACATCTACC
Atf2b		GAATTCGGGATCCGGCA
Atm1a	At1g03680	AATTCTAGGGTTTCCCGATTACG
Atm1b		GAGTCCCATGTTGAATCGTTGA
Atm2a	At4g03520	TCTCCGGCTTCGTTGACC
Atm2b		GAGCTTCACAGACGACGGCT
Atm3a	At2g15570	TCGATTTCACACAGCTCGAC
Atm3b		TTATCCTGTGGACCATCCGAC
Atm4a	At1g50320	AATCGCTCGCGGTGGAC
Atm4b		GATTTGGTACTTCGACGGCG

Table S2. List of identified proteins in *trxf1*, *trxf2, trxm1*, *trxm2, trxm3, and trxm4* mutants**.**

Spot	Mutants analysis	ID	AGI	Protein name	Sequence	Protein MW/PI	Biological Process	Peptide count	Mascot Scores	
									Protein Score	Protein Score C.I. %
**2**	*trxf1/trxf2*	Q0WLF6	At1g78900	V-type ATP synthase	LYDDLNAGFR	69111 Da 5.11	ATP hydrolysis	22	264	100
**3**	*trxf1/trxf2*	Q9SE50	At1g52400	ß-D-glucopyranosyl abscisa ß-glucosidase	GSTDYVGMNYYTSV FAK	60877 Da 6.74	ABA signalling	9	63	96.614
**4**	*trxf1/trxf2*	A4QKH7	AtCg00120	ATP synthase subunit α	TNKPQFQEIIASTK	55305 Da 5.18	ATP synthesis	23	828	100
**5**	*trxf1/trxf2*	O04771	AtCg00490	Ribulose bisphosphate carboxylase large chain	DNGLLLHIHR	47992.2 Da 6.13	Calvin-Benson cycle	20	361	100
**6**	*trxf1/trxf2*	P17745	At4g20360	Elongation factor Tu	ILDEALAGDNVGLLLR	51882.7 Da 5.84	Protein biosynthesis	15	602	100
**7**	*trxf1/trxf2*	O043309	At3g16470	Jacalin-Related lectin	TSQPFGLTSGEEAELG GGK	48523.7 Da 5.12	Stress response	11	319	100
**8**	*trxf1/trxf2*	O65396	At1g11860	Aminomethyltransferase	SLLALQGPLAAPVLQ HLTK	44758.9 Da 8.55	Glycine catabolism	18	443	100
**9**	*trxf1/trxf2*	O82660	At5g23120	Photosystem II stability/assembly factor HCF136	GTGITEEFEEVPVQSR	44133.4 Da 6.79	Photosynthesis	18	493	100

Spot	Mutants analysis	ID	AGI	Protein name	Sequence	Protein MW/PI	Biological Process	Peptide count	Mascot Scores	
									Protein Score	Protein Score C.I. %
**10**	*trxf1/trxf2*	Q9SA65	At1g03050	Putative clathrin assembly protein	SELGGGFDMLLLNG MYQHGAVNAAVK	67271 Da 4.84	Endocytosis	9	39	0
**12**	*trxf1/trxf2*	Q94BN4	At3g03780	5-methyltetrahydropteroyltrigluta-mate-homocysteine methyltransferase 2	YLFAGVVDGR	84899.8 Da 6.09	L-methionine synthesis	7	41	99.079
**13**	*trxf1/trxf2*	P21240	At1g55490	Chaperonin 60 subunit ß1	GYISPYFVTDSEK	64168.8 Da 6.21	Refolding activity	18	206	100
**15**	*trxf1/trxf2*	P19366	AtCg00480	ATP synthase subunit ß	FVQAGSEVSALLGR	53957.1 Da 5.38	ATP synthesis	20	544	100
**16**	*trxf1/trxf2*	Q9LR30	At1g23310	Glutamate-glyoxylate aminotransferase 1	DGYPSDPELIFLTDGA SK	53780.2 Da 6.49	Glycine biosynthesis	21	438	100
**17**	*trxf1/trxf2*	F4JZ46	At5g66190	Ferredoxin-NADP reductase, leaf isozyme 1	DPNATIIMLGTGTGIA PFR	29893.1 Da 5.91	Photosynthesis	8	70	99.259
**19**	*trxf1/trxf2*	Q9ZU25	At1g51980	Probable mitochondrial-processing peptidase subunit α-1	VAEPLTSDLPNVPPQ LAP	54538.8 Da 5.94	Protein processing	8	54	76.029
**20**	*trxf1/trxf2*	Q8RWV0	At3g60750	Transketolase1	VTTTIGYGSPNK	80374.4 Da 5.94	Calvin-Benson cycle	11	271	100
**21**	*trxf1/trxf2*	Q9S7E9	At1g70580	Glutamate-glyoxylate aminotransferase 2	LVLLGDEVYQQNIYQ DER PFISSK	53980.2 Da 6.21	L-alanine degradation	13	91	99.995

Spot	Mutants analysis	ID	AGI	Protein name	Sequence	Protein MW/PI	Biological Process	Peptide count	Mascot Scores	
									Protein Score	Protein Score C.I. %
**22**	*trxf1/trxf2*	Q9C5C2	At5g25980	Myrosinase 2	TIIDDFKDYADLCFER	63262.3 Da 7.14	ABA signalling	10	100	99.999
**23**	*trxf1/trxf2*	Q9SA52	At1g09340[†]	Chloroplast stem-loop binding protein of 41 kDa b	AGGFPEPEIVHYNPK	42764.1 Da 8.19	Polysaccharide degradation	7	54	71.181
**24**	*trxf1/trxf2*	P56757	AtCg00120	ATP synthase subunit α	EQHTLIIYDDLSK	55351 Da 5.19	ATP synthesis	18	773	100
**25**	*trxf1/trxf2*	P92549	AtMg01190	ATP synthase subunit 1	EAFPGDVFYLHSR	55295.9 Da 6.23	ATP synthesis	18	255	100
**26**	*trxf1/trxf2*	B9DGZ4	At1g13440	Glyceraldehyde-3-phosphate dehydrogenase GAPC2	IGINGFGR	36962.1 Da 6.67	Glycolysis	9	157	100
**27**	*trxf1/trxf2*	P10896	At2g39730	Ribulose bisphosphate carboxylase/oxygenase activase	VQLAETYSQAALGDANADAIRGR	52347 Da 5.87	Calvin-Benson cycle	20	506	100
**29**	*trxf1/trxf2*	F4I576	At1g63940	Monodehydroascorbate reductase	EAYAPYERPALTK	52368 Da 7.62	Stress response	16	184	100
**30**	*trxf1/trxf2*	O82660	At5g23120	Photosystem II stability/assembly factor HCF136	GTGITEEFEEVPVQSR	44133.4 Da 6.79	PSII biogenesis	7	94	99.997
**31**	*trxf1/trxf2*	P48491	At3g55440	Triosephosphate isomerase	IIYGGSVNGGNCK	27380.1 Da 5.39	Glycolisis	7	106	100

Spot	Mutants analysis	ID	AGI	Protein name	Sequence	Protein MW/PI	Biological Process	Peptide count	Mascot Scores	
									Protein Score	Protein Score C.I. %
**33**	*trxf1/trxf2*	Q6KCR2	At5g17920	5-methyltetrahydropteroyltriglutamate—homocysteine methyltransferase 1	YGAGIGPGVYDIHSPR	84603.6 Da 6.09	L-methionine biosynthesis	21	488	100
**35**	*trxf1/trxf2*	Q9ZP06	At1g53240	Malate dehydrogenase 1	ALEGADLVIIPAGVPR	36010 Da 8.54	Tricarboxylic acid cycle	6	133	100
**28**	*trxm1/trxm2/trxm4*	O03042	AtCg00490	Ribulose bisphosphate carboxylase large chain	TFQGPPHGIQVER	53434.8 Da 5.88	Calvin-Benson cycle	17	284	100
**37**	*trxm1/trxm2/trxm4*	Q9FI56	At5g50920	Chaperone protein ClpC1	GSGFVAVEIPFTPR	103616.2 Da 6.36	Protein transport	20	147	100
**38**	*trxm1/trxm2/trxm4*	F4JBY2	At3g60750	Transketolase	SIGINSFGASAPAPLLYK	80245.4 Da 5.99	Calvin-Benson cycle	13	86	99.983
**40**	*trxm1/trxm2/trxm4*	O80934	At2g37660	Uncharacterized protein At2g37660	INGEDEVFIGDIR	34972.2 Da 8.37	Response to biotic stress	10	199	100
**41**	*trxm1/trxm2/trxm4*	Q9S841	At3g50820	Oxygen-evolving enhancer protein 1-2	GTGTANQCPTIDGGSETFSFK	35225.8 Da 5.92	PSII stabilization	17	787	100
**42**	*trxm1/trxm2/trxm4*	P0CJ48	At1g29920	Chlorophyll a-b binding protein 2	NRELEVIHSR	28266.2 Da 5.29	Photosynthesis	7	202	100
**43**	*trxm1/trxm2/trxm4*	B9DI26	At5g66190[†]	Ferredoxin-NADP reductase, leaf isozyme 1	LYSIASSAIGDFGDSK	28979.5 Da 5.25	Photosynthesis	11	236	100
**45**	*trxm1/trxm2/trxm4*	O50008	At5g17920	5-methyltetrahydropteroyltriglutamate—homocysteine methyltransferase 1	YGAGIGPGVYDIHSPR	84645.6 Da 6.09	L-methionine biosynthesis	30	784	100

Spot	Mutants analysis	ID	AGI	Protein name	Sequence	Protein MW/PI	Biological Process	Peptide count	Mascot Scores	
									Protein Score	Protein Score C.I. %
**46**	*trxm1/trxm2 /trxm4*	O65719	At3g09440	Heat shock 70 kDa protein 3	IINEPTAAAIAYGLDK K	71559.3 Da 4.97	Stress response	16	364	100
**47**	*trxm1/trxm2 /trxm4*	B9DFU6	At3g08590	Probable 2,3-bisphosphoglycerate-independent phosphoglycerate mutase 2	YLVSPPLIDR	600897 Da 5.53	Glycolysis	13	132	100
**49**	*trxm1/trxm2 /trxm4*	P92549	AtMg01190	ATP synthase subunit 1	LTEVLKQPQYAPLPIE K	55295.9 Da 6.23	ATP synthesis	20	601	100
**52**	*trxm1/trxm2 /trxm4*	Q8RV04	At2g35795	Mitochondrial import inner membrane translocase subunit TIM14-1	KVMVANHPDAGGS HFLASK	11989.4 Da 10.24	Protein transport	6	49	14.948
**53**	*trxm1/trxm2 /trxm4*	Q9FX54	At1g13440	Glyceraldehyde-3-phosphate dehydrogenase GAPC2	GILGYTEDDVVSTDF VGDNR	37004.1 Da 6.67	Glycolysis	18	583	100
**54**	*trxm1/trxm2 /trxm4*	B3H4P2	At1g12900	Glyceraldehyde-3-phosphate dehydrogenase GAPA2	GILDVCDEPLVSVDFR	37928.7 Da 7.63	Calvin-Benson cycle	14	280	100
**55**	*trxm1/trxm2 /trxm4*	P42737	At5g14740	ß carbonic anhydrase 2	EKYETNPALYGELAK	30866.5 Da 5.94	Carbon utilization	13	384	100
**56**	*trxm1/trxm2 /trxm4*	Q39141	At2g34420	Chlorophyll a-b binding protein	FGEAVWFK	28093.1 Da 5.28	Photosynthesis	6	180	100
**57**	*trxm1/trxm2 /trxm4*	Q42029	At1g06680†	Oxygen-evolving enhancer protein 2-1	TNTDFLPYNGDGFK	28249 Da 6.9	Photosynthesis	7	230	100

Spot	Mutants analysis	ID	AGI	Protein name	Sequence	Protein MW/PI	Biological Process	Peptide count	Mascot Scores	
									Protein Score	Protein Score C.I. %
**59**	*trxm1/trxm2/trxm4*	Q944G9	At4g38970	Fructose-bisphosphate aldolase 2	TVVSIPNGPSALAVK	43132.1 Da 6.78	Calvin-Benson cycle	10	82	99.952
**60**	*trxm1/trxm2/trxm4*	Q9T0J9	At4g13280	(Z)-γ-bisabolene synthase 1	EMLEELNTLVRANLDLVK	64151.9 Da 5.37	Secondary metabolism	12	58	88.26
**61**	*trxm1/trxm2/trxm4*	Q944M2	At4g14500	Polyketide cyclase/dehydrase and lipid transport superfamily protein	GDGQQTACEVSLVHYEDMGIPK	49809.1 Da 9.08	Unkown	12	67	98.588
**64**	*trxm1/trxm2/trxm4*	Q9SA52	At1g09340	Chloroplast stem-loop binding protein of 41 kDa b	QLPGESDQDFADFSSK	42764.1 Da 8.19	Carbohydrate metabolism	15	448	100
**65**	*trxm1/trxm2/trxm4*	Q0WLM1	At2g39730	Ribulose bisphosphate carboxylase/oxygenase activase	VQLAETYLSQAALGDANADAIGR	29225.3 Da 4.83	Calvin-Benson cycle	3	62	95.432
**66**	*trxm1/trxm2/trxm4*	Q9S7C0	At1g79930	Heat shock 70 kDa protein 14	GSVIDQLGYCINSYREAAMSTDPK	92490.6 Da 5.15	Stress response	12	53	66.911
**67**	*trxm1/trxm2/trxm4*	O82660	At5g23120[†]	Photosystem II stability/assembly factor HCF136	GTGITEEFEEVPVQSR	44133.4 Da 6.79	PSII biogenesis	17	435	100
**68**	*trxm1/trxm2/trxm4*	P25857	At1g42970	Glyceraldehyde-3-phosphate dehydrogenase GAPB	GILDVCDAPLVSVDFR	48085.7 Da 6.33	Calvin-Benson cycle	10	70	99.398
**69**	*trxm1/trxm2/trxm4*	Q9S9J1	At1g65110	Ubiquitin carboxyl-terminal hydrolase-related protein	TGGCGDINFVHHTISRCPPIFTIVLEWVK	135897.7 Da 6.66	Protein deubiquitination	16	60	92.761

Spot	Mutants analysis	ID	AGI	Protein name	Sequence	Protein MW/PI	Biological Process	Peptide count	Mascot Scores	
									Protein Score	Protein Score C.I. %
**70**	*trxm1/trxm2 /trxm4*	Q0WUV6	At3g52880	Monodehydroascorbate reductase 1	YQTLIIATGSTVLR	50413.4 Da 8.31	Stress response	4	74	99.698
**72**	*trxm1/trxm2 /trxm4*	B3H4P2	At1g12900	Glyceraldehyde-3-phosphate dehydrogenase GAPA2	TFAEEVNAAFR	37928.7 Da 7.63	Calvin-Benson cycle	11	225	100
**73**	*trxm1/trxm2 /trxm4*	Q9LYB4	At3g63080	Probable glutathione peroxidase 5	YGTTVSPLSIQKDIEK	19486 Da 9.28	Stress response	7	52	62.009
**74**	*trxm1/trxm2 /trxm4*	B3H6G1	At5g05113	Uncharacterized protein	HVDWMVRRPVFPSK	10379.5 Da 10.33	Unkown	8	63	96.912
**75**	*trxm1/trxm2 /trxm4*	Q0WVE0	At5g40060	Disease resistance protein (NBS-LRR class) family	ATGNSLVIPLVPTSISL DFLGFR	111004 Da 8.18	Unkown	15	62	95.638
**76**	*trxm3*	P38418	At3g45140	Lipoxygenase 2	LQYLEGVIDER	102552.2 Da 5.43	JA signalling	24	383	100
**77**	*trxm3*	Q9C5C2	At5g25980	Myrosinase 2	TIIDDFKDYADLCFER	63262.3 Da 7.14	ABA signalling	13	203	100
**82**	*trxm3*	G1C2Z0	AtMG01190	ATP synthase subunit 1	EAFPGDVFYLHSR	55221.8 Da 6.23	ATP synthesis	16	221	100
**83**	*trxm3*	B9DGR6	At1g63940	Monodehydroascorbate reductase	VFEYEGSPR	52754.2 Da 7.05	Stress response	12	143	100
**84**	*trxm3*	Q8RY71	At1g54040	Epithiospecifier protein	LGEEGAPAIPR	37042.3 Da 5.56	JA response	13	226	100

Spot	Mutants analysis	ID	AGI	Protein name	Sequence	Protein MW/PI	Biological Process	Peptide count	Mascot Scores	
									Protein Score	Protein Score C.I. %
**85**	*trxm3*	Q9SUI9	At4g04640	ATP synthase γ chain 1	SEPVIHTLLPLSPK	33474.9 Da 6.13	Photosynthesis	11	281	100
**86**	*trxm3*	F4I1X8	At1g55830	Flavin-containing monooxygenase	KPGGSYLEQLNQQLVVK	57189.8 Da 4.93	Unkown	10	54	75.471
**87**	*trxm3*	Q9LJE4	At3g13470	Chaperonin 60 subunit ß 2	DLVGVLEDAIR	63701.5 Da 5.6	Refolding activity	12	118	100
**88**	*trxm3*	F4JCM6	At3g33530	Transducin family protein / WD-40 repeat family protein	ISSPTLDSGADEVAMPSK	149477.4 Da 5.93	Unkown	16	61	94.756
**90**	*trxm3*	F4IVZ7	At2g39730	Ribulose bisphosphate carboxylase/oxygenase activase	GLAYDTSDDQQDITR	48754.5 Da 7.55	Calvin-Benson-Benson cycle	20	280	100
**91**	*trxm3*	Q944G9	At4g38970	Fructose-bisphosphate aldolase 2	YAAISQDSGLVPIVEPEILLDGEHDIDR	43132.1 Da 6.78	Calvin-Benson cycle	14	176	100
**92**	*trxm3*	P42737	At5g14740	ß carbonic anhydrase 2	YAGVGAAIEYAVLHLK	28668.5 Da 5.36	Carbon utilization	14	338	100
**93**	*trxm1/trxm2/trxm4*	P19366	AtCg00480	ATP synthase subunit beta	FVQAGSEVSALLGR	53957.1 Da 5.38	Photosynthesis	21	591	100
**96**	*trxm1/trxm2/trxm4*	Q9LJE4	At3g13470	Chaperonin 60 subunit beta 2	DLVGVLEDAIR	63701.5 Da 5.6	Protein refolding	13	152	100
**97**	*trxm1/trxm2/trxm4*	Q9LR30	At1g23310	Glutamate-glyoxylate aminotransferase 1	GGYFEMTNLPPR	53780.2 Da 6.49	L-alanine degradation	16	153	100

Spot	Mutants analysis	ID	AGI	Protein name	Sequence	Protein MW/PI	Biological Process	Peptide count	Mascot Scores	
									Protein Score	Protein Score C.I. %
**98**	*trxm1/trxm2/trxm4*	Q6KCR2	At5g17920	5-methyltetrahydropteroyltrigluta-mate homocysteine methyltransferase 1	YLFAGVVDGR	84603.6 Da 6.09	L-methionine biosynthesis	24	658	100
**99**	*trxm1/trxm2/trxm4*	Q84R07	At1g56075	Elongation factor 2	AYLPVVESFGFSSQLR	74682.5 Da 6.9	Protein biosynthesis	10	60	93.839
**100**	*trxm1/trxm2/trxm4*	Q93Z12	At4g33010	Glycine dehydrogenase (decarboxylating) 1	VHGLAGIFSLGLNK	75746.7 Da 6.05	Glycine catabolism	12	223	100
**101**	*trxm1/trxm2/trxm4*	Q9SZJ5	At4g37930	Serine hydroxymethyltransferase 1	LRHEVEEFAK	57535.4 Da 8.13	Glycine biosynthesis	15	125	100
**103**	*trxm1/trxm2/trxm4*	F4JUJ5	At4g38970	Fructose-bisphosphate aldolase 2	TAAYYQQGAR	41546.5 Da 9.07	Calvin-Benson cycle	8	63	96.454
**104**	*trxm1/trxm2/trxm4*	C0Z2A8	At1g20020	Ferredoxin-NADP reductase, leaf isozyme 2	DPNATVIMLATGTGIAPFR	28903.4 Da 5.74	Photosynthesis	10	94	99.997
**105**	*trxm1/trxm2/trxm4*	Q9LJG3	At3g14210	GDSL esterase/lipase ESM1	ANPNADASAQQAFVTNVINR	44374.3 Da 7.59	Lipid degradation	6	76	99.845
**106**	*trxm1/trxm2/trxm4*	C0Z2Y9	At1g12900	Glyceraldehyde-3-phosphate dehydrogenase GAPA2	TFAEEVNAAFR	37886.7 Da 7.63	Calvin-Benson cycle	4	55	79.598
